# Development and Analysis of a Stable, Reduced Complexity Model Soil Microbiome

**DOI:** 10.3389/fmicb.2020.01987

**Published:** 2020-08-26

**Authors:** Ryan McClure, Dan Naylor, Yuliya Farris, Michelle Davison, Sarah J. Fansler, Kirsten S. Hofmockel, Janet K. Jansson

**Affiliations:** ^1^Biological Sciences Division, Pacific Northwest National Laboratory, Richland, WA, United States; ^2^Department of Ecology, Evolution and Organismal Biology, Iowa State University, Ames, IA, United States

**Keywords:** soil microbiome, consortia, model microbiome, chitin degradation, network

## Abstract

The soil microbiome is central to the cycling of carbon and other nutrients and to the promotion of plant growth. Despite its importance, analysis of the soil microbiome is difficult due to its sheer complexity, with thousands of interacting species. Here, we reduced this complexity by developing model soil microbial consortia that are simpler and more amenable to experimental analysis but still represent important microbial functions of the native soil ecosystem. Samples were collected from an arid grassland soil and microbial communities (consisting mainly of bacterial species) were enriched on agar plates containing chitin as the main carbon source. Chitin was chosen because it is an abundant carbon and nitrogen polymer in soil that often requires the coordinated action of several microorganisms for complete metabolic degradation. Several soil consortia were derived that had tractable richness (30–50 OTUs) with diverse phyla representative of the native soil, including Actinobacteria, Bacteroidetes, Firmicutes, Proteobacteria, and Verrucomicrobia. The resulting consortia could be stored as glycerol or lyophilized stocks at −80°C and revived while retaining community composition, greatly increasing their use as tools for the research community at large. One of the consortia that was particularly stable was chosen as a model soil consortium (MSC-1) for further analysis. MSC-1 species interactions were studied using both pairwise co-cultivation in liquid media and during growth in soil under several perturbations. Co-abundance analyses highlighted interspecies interactions and helped to define keystone species, including Mycobacterium, Rhodococcus, and Rhizobiales taxa. These experiments demonstrate the success of an approach based on naturally enriching a community of interacting species that can be stored, revived, and shared. The knowledge gained from querying these communities and their interactions will enable better understanding of the soil microbiome and the roles these interactions play in this environment.

## Introduction

Soil microorganisms carry out several important ecosystem functions, including cycling of carbon and other nutrients and support of plant growth. The collective phenotypes of interacting species within the soil microbiome, i.e., the metaphenome, are ultimately responsible for any measured soil function, such as respiration (Jansson and Hofmockel, 2018). However, the high diversity of the soil microbiome (Torsvik et al., 1990, 1996; Sandaa et al., 1999; Yang et al., 2017) with thousands of species and myriads of potential interactions between species (Fierer, 2017; Song et al., 2019; Zhang et al., 2019) makes analysis of specific interactions underlying the soil metaphenome difficult to study.

Multi-omics approaches have been applied to analyze bulk soil samples with the aim of predicting soil microbial functions. Metagenomic sequencing has facilitated the ability to predict both taxonomic and functional gene compositions in soil samples (Jansson and Hofmockel, 2018; Taş et al., 2018; Diamond et al., 2019). However, not all genes are expressed at any given time, and DNA extracted from soil can also represent dormant, or dead, populations that are not contributing to any given function. The soil metatranscriptome has been used to determine functions that are expressed by members of the soil microbiome (Tveit et al., 2014; Hultman et al., 2015; Jiang et al., 2016; Peng et al., 2018; Roy Chowdhury et al., 2019). However, these studies generally rely on relatively shallow metatranscriptomes, with a read depth that is insufficient to cover all the members of the community and therefore only queries the most abundant and transcriptionally active species and genes. Similarly, while there have been advances in soil metaproteomics (Keiblinger et al., 2012; Hultman et al., 2015; Callister et al., 2018), the dynamic range and depth of coverage is still limiting for in depth studies of soil microbiomes. Therefore, although multi-omics approaches are promising technologies for assessing functions carried out by interacting members of the bulk soil microbiome, interpretation of specific interspecies interactions is still challenging.

One approach to investigate the high diversity and complexity of the soil microbiome is to develop reduced complexity model consortia. Ideally, a model consortium would have a tractable and reproducible number of species that are amenable to genetic manipulation and that would enable experimental analysis of population dynamics and specific metabolic and signaling interactions between members (Lozano et al., 2019; Zengler et al., 2019). Ultimately, knowledge gained by analysis of these reduced complexity communities should help to reveal details of metabolic and interspecies interactions that may take place in the native soil community. While simplified communities have previously been constructed to represent the soil microbiome (Goldford et al., 2018; Kong et al., 2018; Rodriguez Amor and Dal Bello, 2019) they have primarily been bottom-up approaches, where specific isolated species are added together to form ‘synthetic consortia’ (Kong et al., 2018; Rodriguez Amor and Dal Bello, 2019). However, a bottom-up approach that combines individual isolates requires either *a priori* knowledge of which species interact in the native environment (to inform combinations) or faces the risk of combining species that may not interact in the native habitat, leading to less translational results.

Recently, we described the development of reduced complexity soil consortia that were obtained by enrichment in sterile soil (Zegeye et al., 2019). In that study, a top-down approach using dilution and growth in soil with n-acetyl glucosamine (NAG) as the primary carbon source allowed for the development of naturally enriched consortia of interacting species. Dilution aided in reduction of species richness and the consortia stabilized after ∼3–5 weeks of incubation (Zegeye et al., 2019). However, the resulting consortia were still relatively complex (several hundred species) and individual isolates were not obtained which precluded study of their specific interactions.

Here, we aimed to develop a model soil consortium using a top down approach to select for a tractable number (30–50) of naturally interacting species. We also aimed to have diverse representation from phyla that are found in soil and to isolate the individual members and examine their pairwise interactions. These consortia were obtained by enriching on agar medium containing chitin as the primary carbon and nitrogen substrate. The solid medium helped to retain some physical structure needed to allow for spatial interactions between species. The chitin substrate was used because it is a complex polymer that provides sufficient chemical complexity to increase our chance of establishing metabolic dependencies between species. Chitin is the second most abundant polymer on the planet and a source of both carbon and nitrogen (Elieh-Ali-Komi and Hamblin, 2016). In soil, chitin is primarily introduced as a component of fungal cell walls and insect bodies (Munro and Gow, 2001; Merzendorfer and Zimoch, 2003; Latgé, 2007). Chitin decomposition has been shown to occur as a stepwise process that requires the coordinated enzymatic capabilities of several microbial populations (Keyhani and Roseman, 1997; Baty et al., 2000a, b; Beier and Bertilsson, 2013). Therefore, we hypothesized that chitin enrichment would result in a diverse community of species that were co-dependent on each other for different steps in the chitin degradation process.

To our knowledge, no reproducible, naturally interacting, model soil consortium with more than a few members has yet been developed. Here, we show that the developed model consortium could be stored as reproducible stocks and revived. In addition, the individual members were obtained in isolation to enable examination of specific interspecies interactions. The resulting naturally interacting soil microbial consortium derived in this study thus provides a valuable resource for the broader scientific community.

## Materials and Methods

### Development of Consortia

Soil samples (Warden silt loam) were collected from the Washington State University field site in Prosser, WA as described previously (Zegeye et al., 2019). Three replicate 100 g portions of the field soil were sieved through a 4 mm sieve and added to 250 ml Mason Jars containing either 0, 10, 50 or 100 ppm of powdered chitin from shrimp shells (Sigma). Sterile deionized water was added to establish field capacity (24% soil moisture) and the jars were weighed once a week with water added as needed to ensure that the soil moisture remained constant. The soil samples were incubated for 7 months at 20°C in the dark, with respiration used to monitor bioactivity once a week (Supplementary Figure S1).

**FIGURE 1:**
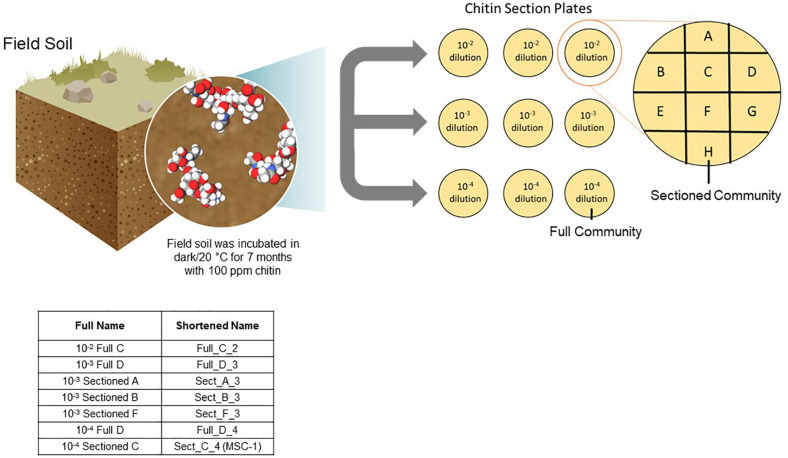
Approach to generating reduced complexity communities. Soil was isolated from an arid grassland field site and incubated with 100 ppm chitin for seven months. Samples of this chitin enriched soil microbiome were collected and diluted to 10^–2^, 10^–3^, and 10^–4^ and plated onto agar plates containing 100 ppm chitin. After one week of growth resulting microbial biomass was either collected from the entire plate and replated onto a new plate (representing a Full community) or sections of the plate were collected separately and replated onto a new plate (representing a Sectioned community). Microbial growth was then replated weekly and amplicon sequenced. Inset table shows the naming of several consortia tested for storage and reproducibility as well as one consortium tested further (MSC-1).

A one-gram sample was collected from the 100 ppm chitin-enriched soil communities, added to 9 mL of Phosphate Buffered Saline (PBS) and 10-fold serially diluted in PBS. Aliquots (100 μL) of the 10^–2^, 10^–3^, and 10^–4^ dilutions were spread onto agar plates containing soil extract and 100 ppm shrimp shell chitin (chitin/soil extract agar). The soil extract was made as described previously (Zegeye et al., 2019), but briefly 500 grams of the field soil was added to 1 L of deionized water and shaken at 160 rpm at 4°C for 48 h. Resulting soil suspensions were centrifuged 30 min at 500 g and the supernatants were filtered using a 0.22 micron filter to produce the soil extract. After one week of incubation at 20°C the collective growth on each plate was re-plated onto a new plate. These represent “Full” communities A-H, where the entire community was re-plated. To enrich “Sectioned” communities, a 100 uL aliquot of each dilution was spread onto a full chitin/soil extract agar plate. After one week of growth at 20°C this plate was divided into 8 sections and each section was re-plated onto a new plate (Sectioned communities A–H) (Figure 1). Both full and sectioned plating approaches were employed to explore possible heterogeneity in the content and structure of developing communities across the agar surface. The communities were re-plated weekly for 22 weeks, with samples collected for amplicon analysis every week for the first 8 weeks.

To store the resulting consortia, microbial biomass was collected from the plate, resuspended in soil extract liquid and the OD was adjusted to 0.35. The cell suspension was diluted 1:10 in soil extract and 25 μL aliquots were added to cryotubes followed by addition of 25 μL of 50% glycerol. To make lyophilized stocks 50 μL aliquots of the 1:10 dilutions were dried for 24 h in a FreeZone 2.5 L, −50°C Benchtop Freeze Dryer Lyophilizer (Labconco). Both glycerol and lyophilized stocks were stored at −80°C. To regrow consortia from stocks, the entire glycerol or lyophilized stock was spread with an inoculum loop onto a 1/4th section of a chitin/soil extract agar plate and incubated at 20°C for one week.

### DNA Extraction and Amplicon Analysis

DNA was extracted from the samples using the DNeasy PowerSoil Kit (Qiagen, Carlsbad, CA). Amplicon analysis was carried out as described previously (Zegeye et al., 2019). Briefly, sequencing was performed on an Illumina MiSeq with 16S primers targeting the V4 hypervariable region of the 16S small-subunit (SSU) rRNA gene using the V4 forward primer (515F) and V4 reverse primer (806R) (Caporaso et al., 2010). 16S datasets were generated by processing Illumina MiSeq reads through an ‘in house’ Hundo pipeline for amplicon quality control and annotation (Brown et al., 2018). Statistical analyses on 16S datasets were performed using the program R, incorporating the R packages ‘phyloseq’ and ‘vegan.’ Separate objects were created for the individual analyses, which included (1) taxonomic analysis of the original plate communities used to generate consortia, (2) isolate communities generated from dissecting the MSC-1 community, (3) reconstitution of consortia from glycerol or lyophilized stocks, and (4) soil incubations of the MSC-1 consortium under distinct perturbations.

For all analyses, separate phyloseq datasets were generated and normalized through rarefying to a constant sequencing depth to retain as many samples as possible without sacrificing resolution. Sequencing read depths were customized to each phyloseq dataset as follows: 5000 (plate communities), 2278 (isolate communities), 5260 (reconstituted glycerol/lyophilized samples), and 1000 (soil incubation communities). The ‘ggplot’ package was used to generate relative abundance plots. For analyses involving alpha diversity, the specific metrics (Simpson’s evenness, richness/observed OTUs) were calculated using the function ‘estimate_richness’ within phyloseq. Student’s *t*-tests (two-sided) were performed using the ‘t.test’ function within the ‘stats’ package. For distances between original vs. reconstituted samples, Bray-Curtis distance objects were generated using the ‘vegdist’ function within the package ‘vegan.’ Student’s *t*-tests (two-sided) and Wilcox rank-sum tests were performed using the ‘t.test’ and ‘wilcox.test’ functions within the ‘stats’ package. For ordination plots using phyloseq objects, distance objects were generated using the ‘ordinate’ function and plots using the ‘plot_ordination’ function within phyloseq. Indicator species analysis was conducted using the ‘indval’ function within the ‘labdsv’ package. Network tables were generated using custom scripts incorporating the ‘cor’ function in the ‘stats’ package and the ‘graph.adjacency’ and ‘delete.vertices’ functions within the ‘igraph’ package.

### Collection of Isolates From MSC-1 and Co-incubation Experiments

To collect isolates for model soil consortium-1 (MSC-1), the community was plated onto chitin/soil extract agar and incubated at 20°C. As colonies emerged, they were re-plated onto chitin/soil extract agar plates and incubated at 20°C until sufficient biomass was available for amplicon analysis. The resultant colonies were isolated, DNA was collected and 16S amplicon analysis was used to putatively identify isolates. Glycerol stocks were made of all isolates by resuspending isolated colonies in sterile soil extract liquid and adding glycerol to a final concentration of 25% before placing in a −80°C freezer for storage. The MSC-1 consortium was also grown on other media to collect additional axenic constituent strains. The additional media included: (1) R2A agar (Reasoner and Geldreich, 1985), (2) agar with 100 ppm chitin and 10% soil extract (diluted in water), (3) agar made with 100 ppm chitin and no soil extract (replacing with water), and (4) agar made with soil extract but no chitin.

For monitoring of interspecies interactions, isolates were grown on R2A agar and resulting microbial growth was resuspended in minimal liquid medium (M9) (Harwood and Cutting, 1990) to a final OD of 0.1. A 500 μL aliquot for each strain was added to wells of a 96 well plates in triplicate to determine axenic growth rates. For growth of co-cultures, 250 μL of each strain to be co-cultured was added to the same wells in triplicate. The chitin monomer, n-acetyl glucosamine (NAG), was then added to all wells to a final concentration of 10 mM. Plates were placed into a Synergy Neo2Hybrid Multi-Mode Reader (Biotek) and cultured with shaking at 24°C for 5 days with OD collected from each well every 10 min. To determine if co-culturing led to probable positive or negative interactions, we compared the expected OD of a pairwise co-culture (Expected_OD = OD of Monoculture of Species A^∗^0.5 + OD of Monoculture of Species B^∗^0.5) to the observed OD of this co-culture. The expected OD is derived from half the OD’s of each constituent species of that pair because each species made up half of the volume in the well. If the observed OD of the co-culture in the plate reader was higher than the expected OD of each constituent pair added together this suggests a positive interaction. If the expected OD is lower than the observed OD this suggests a negative interaction.

### Incubation of MSC-1 in Soil and Network Analysis

MSC-1 was grown on chitin/soil extract agar for one week at 20°C. Resulting MSC-1 growth was collected and resuspended in soil extract to an OD of 0.35. This resupension was then diluted 1:10 using soil extract and 800 μL of this dilution was added to 8 g of sterile field soil (sterilized by autoclaving and pre-wet with 1.2 mL soil extract 48 h before the start of the experiments). The following treatments were applied to the sterile soil incubations: (1) Standard: sterile soil wet to 25% (v/v) with soil extract, incubated at 20°C with 100 ppm chitin; (2) high temperature (HighTemp): same as Standard but incubated at 37°C; (3) Low Temperature (LowTemp): same as Standard but incubated at 10°C; (4) No chitin (nochitin): same as Standard but lacking chitin; (5) Salt stress (SaltStress): same as standard but supplemented with 100 mM NaCl; (6) incubation with 2,4-Dichlorophenoxyacetic acid (Herbicide): same as Standard but supplemented with 0.015 mg of 2,4-Dichlorophenoxyacetic acid per gram of soil. Each sterile soil incubation condition was performed with 5 replicates. Sub-samples (0.25 g) were collected once a week for amplicon analysis using the approach described above.

To determine species co-abundance networks, Pearson correlation coefficients were used to calculate co-abundances among species using abundance data from all replicates, perturbations and timepoints. Edges in the resulting networks reflect instances of correlation that are higher than 0.35 or lower than −0.35. Nodes represent OTUs. Networks were viewed in Cytoscape which was also used to calculate betweenness values (Shannon et al., 2003).

## Results

### Development of Model Soil Consortia

Soil was previously enriched for 7 months using chitin as a substrate and respiration was monitored (Figure 1). This soil was inoculated onto chitin/soil extract agar plates to encourage interspecies cross-feeding and metabolic interdependencies needed for growth on the chitin substrate. Microbial growth that was collected from a full chitin/soil extract agar plate was designated a “Full” community, while growth on a defined 1/8th section of the plate was designated a “Sectioned” community (Figure 1) to capture potential heterogeneity of growth of the soil community on the agar surface. We generated eight “Full” and eight “Sectioned” communities (A–H); each with several starting soil inoculum dilutions. Each community was then re-plated weekly over 22 weeks to allow for the resident microbial populations to establish metabolic dependencies and stable community compositions as determined by amplicon sequencing.

**FIGURE 2:**
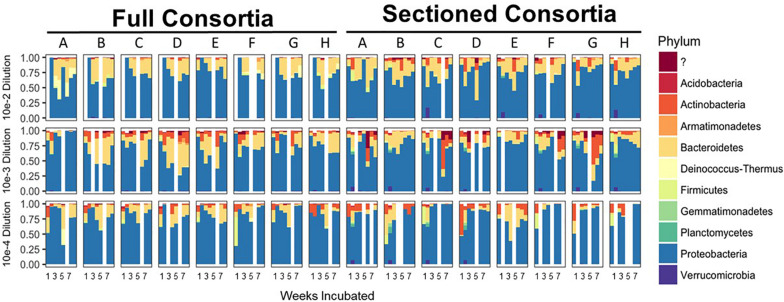
Enrichment of reduced complexity communities on chitin/soil extract agar medium. Relative abundances for each microbial community at the phylum level are shown on the x-axes with communities from the 10^–2^ dilution on the top, 10^–3^ dilution in the middle, and 10^–4^ dilution on the bottom. Full and Sectioned communities from the agar medium are, respectively, labeled and the weeks of incubation are shown on the y-axes.

After 8 consecutive weeks of re-plating both Full and Sectioned consortia, 16S amplicon analysis was used to determine the bacterial constituents of the emerging consortia as well as their richness and evenness. In contrast to what we and others have previously demonstrated with liquid cultures (Goldford et al., 2018; Zegeye et al., 2019), culturing on solid medium led to consortia that were more diverse at the phylum level, with few consortia that were completely dominated by Proteobacteria/Pseudomonads. Proteobacteria was still, however, the major phylum in most of the consortia, followed by Bacteroidetes and Actinobacteria. These same three phyla were dominant in the native source soil (Zegeye et al., 2019) showing that these consortia are representative of the native soil. Several consortia also contained minority populations of Firmicutes, Gemmatimonadetes, and Planctomycetes (Figure 2). Many consortia had blooms of similar species at specific time points. For example, several of the Full communities at the 10^–2^ dilution showed an initial bloom of Proteobacteria followed by emergence of other phyla (primarily Bacteroidetes) before they began to stabilize. In addition, many Sectioned consortia, showed a small but very consistent bloom of Verrucomicrobia after two weeks of incubation. Sectioned consortia were also generally more diverse than Full consortia with higher amounts of Actinobacteria.

**FIGURE 3:**
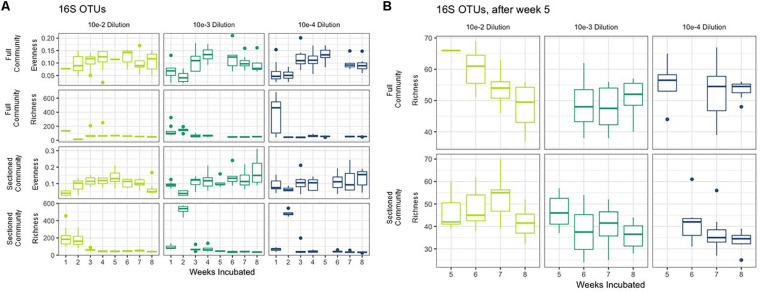
Richness and evenness of Consortia. **(A)** Average richness and evenness levels for Full and Sectioned communities at the three dilution levels are shown on the x-axes. Weeks incubated are shown on the y-axes. **(B)** A zoom in of richness levels from weeks 5–8 for ease of viewing.

We successfully obtained consortia that had a greatly reduced complexity compared to the native soil (which had >750 OTUs after rarefaction). All of the consortia had fewer than 100 OTUs after 8 weeks of growth, with most containing between 30–70 OTUs (Figures 3A,B). As with taxonomic content, there was a temporal element to the reduction in species richness. After 2 weeks, two sets of consortia (Sectioned consortia diluted to 10^–3^ and Sectioned consortia diluted to 10^–4^) had a high richness, with an average of 550 (+/- 75) and 475 (+/- 25) OTUs, respectively. However, richness rapidly dropped after week 2 and more slowly at later timepoints (weeks 5–8). Interestingly, there were differences when comparing Full to Sectioned consortia. Sectioned consortia increased in richness at week 2 (see above), which was higher than that observed at week 1 (100 +/- 20 OTUs). Week 2 was also the timepoint when a bloom of Verrucomicrobia emerged, along with a bloom of Firmicutes, Gemmatimonadetes and Planctomycetes. This increase in species richness, as well as the bloom of these additional phyla, was confined only to Sectioned communities that were diluted to 10^–3^ and 10^–4^ but was very consistent across these communities. Sectioned communities also showed a stronger response to dilution when examining species richness. Sectioned communities that were derived from 10^–2^ dilutions contained between 40–55 OTUs, 10^–3^ consortia between 35–45 OTUs and 10^–4^ consortia approximately 30–40 OTUs (Figure 3B). By contrast, we did not detect decreases in species richness of Full communities as a result of dilution; at 5–8 weeks 10^–2^ consortia contained between 45–65 OTUs, 10^–3^ consortia between 45–55 OTUs and 10^–4^ consortia approximately 55 OTUs.

### Storage and Reconstitution of Consortia

**FIGURE 4:**
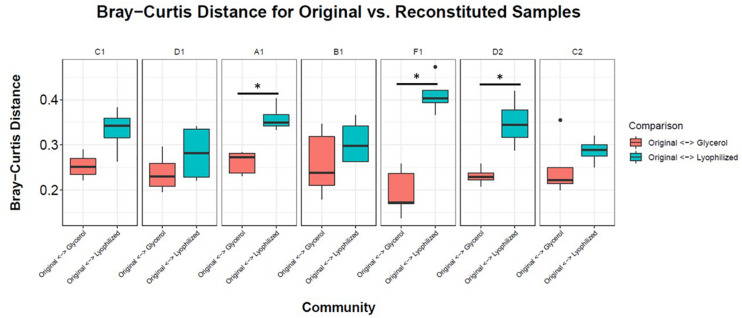
Storage and reconstitution of consortia. Box and whisker plots showing the average Bray-Curtis distance of 4–5 replicates of reconstituted glycerol (red boxes) or lyophilized (blue boxes) stocks compared to the respective parent consortium on the plate. Asterisks indicate statistically significant differences (<0.03) between glycerol or lyophilized stocks by Wilcox rank sum test.

To facilitate sharing our consortia as a resource for the research community, the consortia were then tested for their ability to be stored either as glycerol stocks or as lyophilized stocks at −80°C, and how well they could be revived after storage. After 22 weeks of weekly re-plating, seven different consortia were collected from plates and either frozen in glycerol stocks or lyophilized (Figure 1). Consortia were chosen based on (1) having a high diversity of phyla, (2) containing taxa of interest and (3) representing both Full and Sectioned consortia from all three dilution levels so we could explore how each type of consortia (Full vs. Sectioned) and dilution (10^–2^ vs. 10^–3^ vs. 10^–4^) might affect storage and revival. It is noteworthy that even after 22 weeks of consecutive re-plating most of the consortia still had a diverse representation of phyla (Supplementary Figures S2–S4 and Supplementary Table S1). For all consortia the Bray-Curtis distances between reconstituted glycerol stocks and the parent consortia (average 0.241 ± 0.022) were consistently less than the distances between reconstituted lyophilized stocks and the parent consortia (average 0.332 ± 0.046) (Figure 4 and Supplementary Table S2). For three of the consortia (Sect_A_3, Sect_F_3, and Full_D_4) not only were glycerol stocks closer to the parent but the Bray-Curtis difference between glycerol and lyophilized stocks was statistically significant (*p* < 0.03) (Supplementary Table S2). Only consortia Full_C_2 stored using glycerol and Full_D_3 stored using lyophilization showed significant differences in taxonomic membership between the parent consortium and its stocks (Supplementary Figures S5, S6). However, with the exception of glycerol stocks of Full_D_3 and lyophilized stocks of Full_D_4 and Sect_A_3, there was a significant difference (*p* < 0.05) between stocks of a given consortium and parent stocks of the other six consortia tested (Supplementary Figures S5, S6), illustrating that not only are stocks representative of their parent consortia, they are also dissimilar from other parent consortia. Despite the general fidelity of storage and reconstitution there were some shifts with certain consortia as a function of storage and freezing. All OTUs and their shifts in abundance as a function of freezing after glycerol or lyophilization storage are shown in Supplementary Table S1.

**FIGURE 5:**
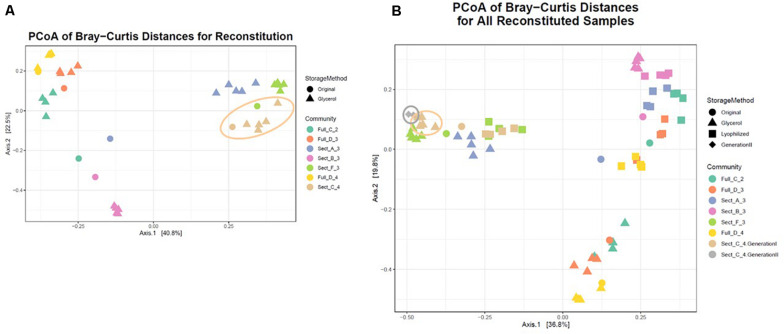
Bray-Curtis distances of all consortia and reconstituted stocks. **(A)** Each parent consortium is shown as a colored circle, colors for each consortium are shown on the right. Each glycerol stock is shown as a colored triangle with colors that match its parent consortium. Consortium C2 (the origin of MSC-1) is indicated with a beige outline. **(B)** Similar to **(A)** but lyophilized stocks of each consortium are also included (colored squares). Also shown is Generation 2 of C2 (gray diamonds). Generation 1 of C2 is indicated with a beige outline and Generation 2 of C2 is indicated with a gray outline.

A single consortium, Sect_C_4 (Figure 5 and Table 1), was subsequently chosen to focus on for further analysis and to develop as a model soil consortium for the larger research community. This consortium was chosen because (1) it could be successfully revived from either glycerol or lyophilized stocks stored at −80° C, (2) it had a relatively low complexity with 35 bacterial OTUs that were detected by 16S amplicon sequencing (3) it had a higher diversity and evenness at the phylum level compared to the other six consortia and (4) it contained OTUs of interest that were found in the source soil, including representatives matching taxa known to respond to or degrade chitin such as Rhodococcus (Sun et al., 2014) and Streptomyces (Schrempf, 1999, 2001). We also detected fungal species within this consortium using ITS sequencing: one unknown fungal species as well as a species in the family Mycosphaerellaceae (Supplementary Table S3), a family of fungi that is known to have a number of interactions with plant species (Aguilera-Cogley et al., 2017). Importantly, a second round of storage and revival (Generation 2) had little impact on the makeup of this consortium (Figure 5B and Supplementary Figure S7). This consortium was re-designated: Model Soil Consortium – 1 (MSC-1).

**TABLE 1:** Taxonomic makeup of MSC-1.

OTU_ID	Phylum	Order	Family	Genus	Counts	Isolate matches based on V4 region
OTU_7	Actinobacteria	Corynebacteriales	Nocardiaceae	Rhodococcus	1803	Rhodococcus Isolate
OTU_8	Bacteroidetes	Chitinophagales	Chitinophagaceae	Taibaiella	602	
OTU_11	Bacteroidetes	Cytophagales	Cytophagaceae	Dyadobacter	539	Dyadobacter Isolate
OTU_9	Proteobacteria	Sphingomonadales	Sphingomonadaceae	Sphingopyxis	350	
OTU_6	?	?	?	?	325	
OTU_27	Proteobacteria	Rhizobiales	Phyllobacteriaceae	?	254	
OTU_22	Proteobacteria	Rhizobiales	Bradyrhizobiaceae	Bosea	244	
OTU_5	Actinobacteria	Streptomycetales	Streptomycetaceae	Streptomyces	238	Streptomyces Isolate
OTU_2	Proteobacteria	Burkholderiales	Comamonadaceae	Variovorax	217	Variovorax Isolate
OTU_4	Proteobacteria	Rhizobiales	Rhizobiaceae	Ensifer	205	Ensifer Isolate*
OTU_13	Proteobacteria	Rhizobiales	Rhizobiaceae	Ensifer	190	Ensifer Isolate*
OTU_23	Proteobacteria	Rhodospirillales	Rhodospirillaceae	Inquilinus	164	
OTU_36	Proteobacteria	Rhizobiales	Xanthobacteraceae	?	43	
OTU_25	Proteobacteria	Burkholderiales	Oxalobacteraceae	Massilia	31	
OTU_30	Proteobacteria	Burkholderiales	Comamonadaceae	?	13	
OTU_16	Proteobacteria	Caulobacterales	Caulobacteraceae	Caulobacter	8	
OTU_64	Proteobacteria	Rhizobiales	Bradyrhizobiaceae	?	7	
OTU_61	Proteobacteria	Rhizobiales	Rhizobiaceae	Rhizobium	6	
OTU_43	Actinobacteria	Corynebacteriales	Mycobacteriaceae	Mycobacterium	6	
OTU_159	Proteobacteria	Rhodocyclales	Rhodocyclaceae	?	2	
OTU_32	Armatimonadetes	Fimbriimonadales	Fimbriimonadaceae	?	2	
OTU_68	Proteobacteria	Rhizobiales	Rhizobiales_Incertae_Sedis	Rhizomicrobium	2	Rhizobium Isolate
OTU_14	Proteobacteria	Caulobacterales	Caulobacteraceae	Brevundimonas	1	
OTU_65	Proteobacteria	Rhizobiales	Rhizobiaceae	Ensifer	1	
OTU_54	Proteobacteria	Rhizobiales	?	?	1	
OTU_17	Actinobacteria	Micrococcales	Micrococcaceae	?	1	
OTU_168	Actinobacteria	Frankiales	Geodermatophilaceae	Blastococcus	1	
OTU_80	Firmicutes	Bacillales	Bacillaceae	Bacillus	1	
OTU_73	Firmicutes	Bacillales	Bacillaceae	Bacillus	1	
OTU_94	Bacteroidetes	Chitinophagales	Chitinophagaceae	?	1	
OTU_113	Proteobacteria	Rhodospirillales	Rhodospirillaceae	Inquilinus	1	

*Based on V4 amplicon data it could not be distinguished which of these Ensifer species matches to the Ensifer isolate.

### Isolation of Constituent Species From MSC-1

**FIGURE 6:**
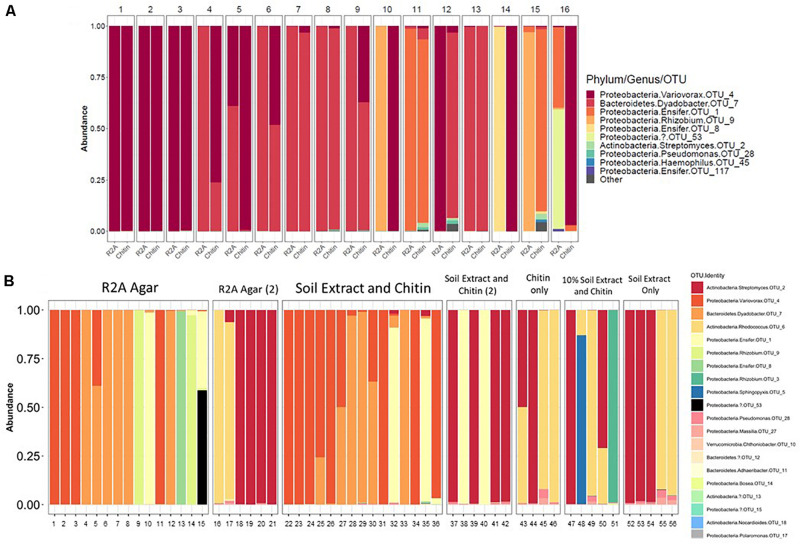
Isolation of microbial constituents of Model Soil Consortium MSC-1. **(A)** MCS-1 isolates were obtained either on chitin/soil extract plates, indicated by “Chitin” or R2A agar plates, indicated by “R2A.” After 16S amplicon sequencing analysis, read counts were rarified to 2278 and the number of counts aligning to different phyla for each colony is shown. **(B)** Similar to **(A)** but a second round of isolation using additional media sources, colonies are not numbered, each column indicates an individual colony subjected to 16S amplicon sequencing analysis. Multiple rounds [indicated by a (2)] were performed for R2A agar and for chitin/soil extract agar.

There were 31 bacterial OTUs found in MSC-1 (Table 1). However, more than 98% of the counts (aligned reads) were represented by the top 13 OTUs. The most abundant OTU was a species of the *Rhodococcus* genus, followed by a *Dyadobacter* and a *Taibaiella* species. Species of the *Bosea, Streptomyces, Ensifer*, and *Variovorax* genera were also abundant as was an unknown bacterium that represented approximately 5% of the reads. Because these species were co-enriched together over a 22-week period and through two rounds of freezing and revival, we hypothesized that there existed metabolic co-dependencies within MSC-1. To better understand these interactions at a pairwise level, individual species were isolated from within MSC-1 for further analysis and characterized by 16S sequencing. A replete medium (R2A) was used to obtain separate isolates of species that might otherwise be metabolically dependent on each other on chitin/soil extract plates. In an initial round of isolation three ‘pure’ strains were isolated as confirmed by 16S analysis (>98% of the reads matched to a single OTU): (1) A *Variovorax* strain isolate, which grew on both R2A medium and chitin/soil extract plate (represented in Figure 6A, Col. 1). (2) A *Dyadobacter* strain isolate that grew well on R2A media (represented Figure 6A Col. 4). Interestingly, when cultured on a chitin/soil extract plate this strain was instead found mainly in colonies that also contained significant amounts of the *Variovorax* strain (Figure 6A, Col. 6, and 9). (3) An *Ensifer* strain isolate that grew in colonies on either R2A medium but not a chitin/soil extract plate (represented in Figure 6A, Col. 14).

A second round of isolation using diverse growth conditions, and longer incubation times enabled isolation of additional strains. These included a species of the *Rhodococcus* genus (represented in Figure 6B, Col. 16) that, in addition to R2A, was able to grow on chitin plates without soil extract, as well as on plates containing only soil extract. In addition, a species in the *Rhizobium* genus (represented in Figure 6B, Col. 51) was also found on plates that contained chitin and a low amount of soil extract (10%). Finally, a species of *Streptomyces* (represented in Figure 6B, Col. 18) was also isolated from several of the other conditions we tested (Figure 6B). None of the isolates were able to be identified beyond genus level using the 16S amplicon data and are therefore identified using OTU numbers (Table 1); note that we will refer to them at the genus level for clarity of presentation throughout. Overall, six separate isolates representing six genera, five families, five orders and three phyla were obtained from MSC-1 (Figures 6A,B). Amplicon matches between OTUs identified in MSC-1 and these 6 six isolates are shown in Table 1.

### Co-culture Analyses of MSC-1 Isolates

**FIGURE 7:**
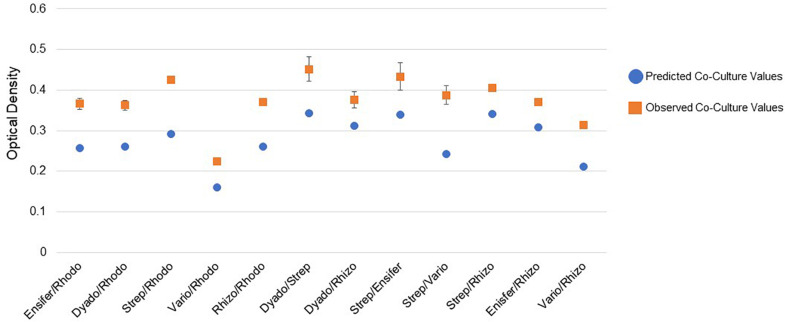
Pairwise interactions of MSC-1 isolates during growth on N-acetyl-glucosamine (NAG). Pairwise combinations of species are shown on the x-axis (Rhodo-Rhodococcus; Dyado-Dyadobacter; Strep-Streptomyces; Vario-Variovorax; Rhizo-Rhizobium). Optical density (OD 600) is shown on the y-axis and represents OD collected during early exponential phase of growth. Blue circles represent predicted co-culture values, orange squares represent observed co-culture values.

Pairwise incubations were carried out using different combinations of the six MSC-1 isolates during growth with NAG as a source of carbon and nitrogen in M9 minimal liquid medium. Here, NAG was chosen due to its being the monomer of chitin and being soluble in water, making in more amenable to OD based growth experiments looking at co-cultures of strains. When examining mono- and co-cultures during early exponential phase we identified several co-cultures that had higher observed ODs than would be predicted from a combination of their ODs when growth in monoculture (see Methods). All of these increases were statistically significant by *t*-test (*p* < 0.01) (Figure 7). Several of the combinations showed much higher growth than would be predicted including *Streptomyces* paired with *Rhodococcus*, *Streptomyces* paired with *Variovorax*, and *Rhizobium* paired with *Rhodococcus*. Paired isolates showing a higher optical density than predicted during co-culture suggest a positive interaction. We also examined mono- and co-cultures by OD during late exponential phase but found that the co-cultures were not significantly higher or lower compared to monocultures at this timepoint (data not shown).

### Analysis of MSC-1 in Sterile Soil

MSC-1 was next studied in the context of its native sterile soil environment under six different growth conditions representing different field perturbations: (1) standard conditions (100 ppm chitin, 20°C), (2) standard conditions, without chitin, (3) high temperature (37°C), (4) low temperature (10°C), (5) herbicide addition (2,4-Dichlorophenoxyacetic acid, 0.0015 g/gram of soil), and (6) salt stress (100 mM NaCl).

**FIGURE 8:**
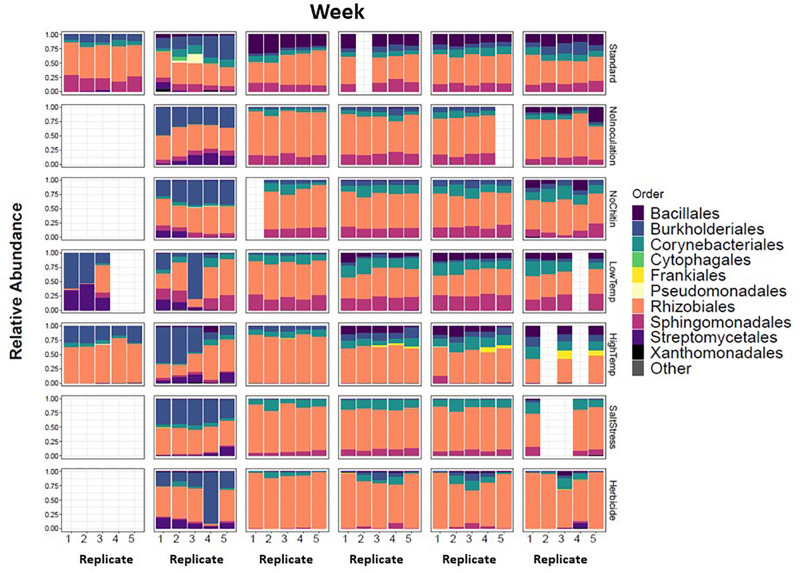
Taxonomic shifts of MSC-1 during growth on chitin in soil. Relative abundance of each replicate during five week incubations is shown on the y-axes, weeks incubated are shown at the top and replicates on the x-axes. Columns are colored by Order, a taxonomic rank used because we now have more reduced complexity community and can look more closely than at the Phylum level used in earlier analyses.

There were major changes in the community composition of MSC-1 during the first week of soil incubations with a relative increase of Burkholderiales in all samples regardless of condition (Figure 8), suggesting the possibility that this taxon responds to cultivation in sterile soil. There were also relative increases of Streptomycetales in several of the soil incubation conditions and replicates, although this was not universal. By week 2 most of these initial changes were no longer apparent. MSC-1 had stabilized and reflected relative abundance profiles that would persist through the end of the experiment at week 5. At that point many of the different incubation conditions had led to statistically significant (*p* < 0.05) changes in the makeup of MSC-1. Indicator species analysis showed that increased temperature led to relative increases of species in the Frankiales and Corynebacteriales orders as well as the Mycobacteriaceae family and separate species in the *Mycobacterium* genus. This same analysis showed that growth without chitin led to increased relative abundance of species in the Bacillales order. Furthermore, growth under low temperature conditions also led to increased relative abundance of species in the Corynebacteriales order as well as separate species in the family Mycobacteriaceae and two additional OTUs: one in the Bradyrhizobiaceae family and the other in the *Bosea* genus (Supplementary Table S4). In addition to these statistically significant changes in community composition, we also observed consistent but statistically insignificant relative increases of Rhizobiales and Corynebacteriales with a corresponding relative decrease in Bacillales and Burkholderiales under salt stress conditions. Finally, growth under 2,4-D incubation conditions generally resulted in a relative increase of Rhizobiales but the response to this condition was more varied across replicates.

**FIGURE 9:**
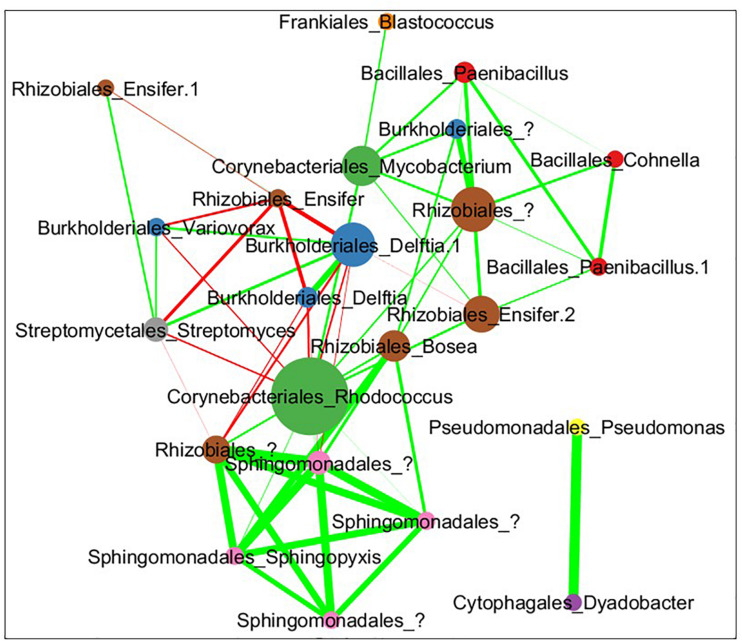
Pearson correlation coefficient network of co-abundance data from MSC-1 in soil. Each circle represents a specific OTU with the name of the species shown as “Order”_“Genus.” These taxonomic levels were chosen because we now have a simple community represented in the network, allowing for more detailed taxonomies to be examined. Nodes are sized by their betweenness centrality in the network, those showing higher betweenness being larger. Lines indicate instances of high correlation coefficient, either >0.35 (green lines) or <0.35 (red lines) thickness of lines represents the strenght of the correlation.

The combined soil data was used to infer a species co-abundance network with the aim of viewing potential interactions between species in the MSC-1 consortium. Normalized species abundance data was used together with Pearson correlation coefficients to infer a network that linked species based on their co-abundance. We also applied centrality analysis (Shannon et al., 2003) to the resulting network to view which species occupied positions of high betweenness, a proxy for their importance to the network and thus to MSC-1 as a whole. This analysis showed that species of the *Rhodococcus*, *Mycobacterium* and *Rhizobiales* genera occupied positions of high centrality in the network (Figure 9). *Rhodococcus* had several positive and negative correlations with other genera of MSC-1, including *Streptomyces*, *Variovorax*, and *Delftia* (negative) and *Rhizobiales* (positive). Several of these correlations agreed with our growth experiments using pairwise species combinations in liquid (Figure 7). For example, observed *Ensifer* and *Rhodococcus* co-culture ODs were higher than predicted suggesting positive interactions (Figure 7). These species were also found to have a positive correlation in our network (Figure 9). The same was found with *Rhizobiales* and *Rhodococcus* as well as *Streptomyces* and an OTU assigned to Ensifer (Proteobacteria_Rhizobiales_Ensifer.1), although another *Ensifer* OTU (Proteobacteria_Rhizobiales_Ensifer) had a negative correlation with *Streptomyces* (Figure 9). However, it should be noted that interactions based on species co-abundance networks (Figure 9) were derived from incubation of MSC-1 in soil with chitin while pairwise interactions (Figure 7) were derived from isolates in liquid media using NAG. While some similarities were found, the differences in these experiments preclude a direct comparison of their results.

## Discussion

The soil microbiome carries out a myriad of biochemical reactions needed to decompose soil organic matter. The fact that interspecies interactions exist in soil relevant to these reactions has been known for decades (Melillo et al., 2017; De Vries et al., 2018), but the details of many of these interactions are not known, nor are the constituent species that participate in them. Here we aimed to develop a model soil consortium that could be leveraged to understand details of metabolic co-dependencies between species during decomposition of a complex carbon substrate. Detailed analyses of a simpler model consortium offers a major advantage over bulk omics analysis of the native soil because of the more tractable community membership in the model community. The use of solid agar medium to isolate microbial species is by no means a new approach, however, here we took advantage of the element of physical structure that can emerge with agar plates (compared, for example, to a free mixing liquid cultivation system) to allow for the development of spatial interactions between naturally interacting members of the soil community. We hypothesized that such a community would be comprised of species that are mutually dependent on each other, especially for metabolism of complex carbon and nitrogen sources, such as chitin. This is supported by the fact that many of the isolated colonies that we grew on plates contained multiple taxa examined via amplicon analysis. This was especially prevalent when grown on chitin plates but was less apparent on R2A plates. As R2A is a rich medium this may allow for more independent growth of each species compared to chitin, which may require more interactions. This is one of the reasons we chose R2A when attempting to obtain isolates. By allowing for a naturally developed community to emerge, as we did here, we selected for those species that participate in such interactions, capturing them for more in depth analysis. To try to select for independencies among constituent species we utilized a complex carbon and nitrogen source, chitin, when we developed consortia. This likely had a role in their development as the ability of the initial plated soil microbiome to degrade chitin would have an effect on how quickly and in which direction the consortia developed. Because this initial species makeup (from plating soil) is stochastic in nature this led to a number of different communities after several weeks of growth. Future experiments may repeat this approach but measure the consumption of chitin (or other complex C and N sources) to track how the community is utilizing these sources and determine whether and to what degree evolved communities are influenced by the nutrient source chosen to evolve them.

A number of studies have looked at synthetic consortia of soil microorganisms that combine previously isolated species to examine interactions among them with a high level of detail (Kong et al., 2014; Rodriguez Amor and Dal Bello, 2019), a bottom-up approach. However, these studies have the disadvantage of developing consortia with few members (leading to only a small part of the soil functional and interaction network being viewed) or with populations that do not naturally interact with each other in the environment. By taking a top-down approach as we do here, we allowed the community to self-select members and enrich for critical interactions needed for growth. This top-down approach has previously been shown to be superior to a bottom-up approach when developing representative environmental communities (Gilmore et al., 2019). In addition, the larger size of this community (35 detected OTUs with ∼13 dominant species, compared to 2–3) means that a greater amount of the soil functional potential and species interactions was captured and examined. An important attribute of this approach is that the community was representative, to a degree, of what is found in the native soil microbiome, containing species from diverse phyla, orders and families. This included the identification of difficult to cultivate organisms such as Verrucomicrobia (Fierer et al., 2013; Choi et al., 2017). It is possible that difficult-to-cultivate species may only emerge in the context of a community, requiring interactions with other members and precluding their growth as axenic strains without more biochemical information. Species that co-isolate together is a phenomenon that has been seen previously (Lozano et al., 2019). These “hitchhikers” have been used to build consortia that have a high probability of containing interacting organisms. Our use of agar here also has the potential to isolate hitchhikers and in fact this may be the case for some isolates under certain conditions such as when *Dyadobacter* co-isolated with a *Variovorax* strain under chitin conditions but not R2A conditions (Figure 6A). Use of a top-down dilution approach, such as what is presented here, may aid in capturing these and other organisms of interest by maintaining such interactions and allowing for their study and identification.

Synthetic communities do have several advantages, including detailed information on isolates, their genomic potentials, and control of species abundances. In addition, their simplicity can be useful in untangling especially complex interactions. We showed here that combining a top-down and bottom-up approach that is focused on the same model community yielded results that take advantage of the strengths of both approaches. By starting with a top-down approach to reduce the soil microbiome to a more manageable size and to identify who the interacting members are, we then used this knowledge to study pairwise comparisons (a simple bottom-up approach) and delineated these interactions with more detail.

The observation that in the initial weeks of consortia development there were large changes in richness and diversity suggests that researchers could isolate several different consortia from the initial growth of a single inoculum by harvesting and storing at different timepoints. However, this approach may not work in all cases. We attempted to capture a soil consortium early in our initial cultivation (week 2) due to the emergence of Verrucomicrobia but found that revival of this same community from a glycerol stock was not possible (data not shown). This suggests that in the early weeks of development consortia may be too unstable for storage. A time gap between initial development of a reduced-complexity consortium and its stabilization was also found in our earlier study (Zegeye et al., 2019). One reason for this lack of initial stability may be that after dilution and plating there is not enough time (in 2 weeks) for species to reach high enough densities to participate in required interactions that stabilize the consortia. Other studies have found that interspecies interactions, including negative interactions, act to stabilize consortia over both the short term (∼36 h) and long term (∼2 weeks) (Kim et al., 2008; Minty et al., 2013). These observations suggest that care must be taken to allow sufficient time for consortia to stabilize (several weeks as we use here though the time required may vary), particularly when using a top-down approach, before they can be stored and revived. It should also be noted that we saw a similar effect when we moved MSC-1 to sterile soil. During the first week of soil incubation there were significant changes in relative abundances of several taxa that later stabilized after 2 weeks. Thus, when studying model consortia it is also advisable to allow for stability to emerge when moving consortia to a new environmental medium for analysis.

Based on our storage and revival results we propose that when evaluating model consortia as a tool for downstream study and dissemination among researchers, two tests should be applied regarding use of the consortia for research. First, it is necessary to confirm that the consortium, after being stored as a stock and re-plated, is taxonomically similar to the parent community. Of the seven consortia we tested this was the case for five of them. Second, it is necessary to confirm that individual reconstituted stocks of the consortia show high similarity to each other. If individual reconstituted stocks show high differences from each other, that would imply that re-growth of the consortium is a stochastic rather than a deterministic process or that unknown selection pressures are present. This would severely curtail the potential of the consortia to serve as a model microbial system for the research community. The second question is likely to be the more important question of the two, because similarity of among stock replicates is critical for sharing with the scientific community, even if an initial shift from the parent consortium is present. Of course, there is no way to guarantee that if the model consortium is shared that it will remain static. Therefore, there would need to be a process in place for routine calibration with the source consortium. It is promising, however, that all of our consortia showed high similarity (Bray-Curtis distances of <0.05) within replicates of their revived glycerol stocks, making them potentially useful tools for other scientists. In addition, while there was some drift between Generation 1 of MSC-1 and Generation 2 (Figure 5 and Supplementary Figure S7) this drift was minimal (these two generations showed a Bray-Curtis distance of ∼0.1). In addition, we took steps to dissect MSC-1 into its constituent species. These could also be shared as separate axenic strains and recombined using a bottom up approach where species are added together to reflect their natural relative abundance in MSC-1.

During analysis of MSC-1 we found that several species occupied central positions in a co-abundance network based on 16S data and were highly abundant in the consortium. These included species of *Mycobacterium, Rhodococcus*, and *Rhizobiales*. As this consortium was enriched under conditions of abundant chitin, we posit that these species are acting together to degrade chitin and provide simpler nutrient sources to other members of MSC-1, but this remains to be confirmed in subsequent research. Some *Rhodococcus* species have been reported to degrade chitin (Sun et al., 2014) and an additional study found that the *Rhodococcus* genus increases in soils amended with chitin (Jacquiod et al., 2013). Therefore, *Rhodococcus* in MSC-1 may also play a role as a primary chitin degrader that provides simpler nutrient sources to other species. This is corroborated by our co-culture work (Figure 7) where several genera of MSC-1 (including *Ensifer*, *Dyadobacter* and *Rhizobium*) showed higher growth when co-cultured with *Rhodococcus*.

Interestingly, some *Rhizobiales* species have been shown to produce modified chitin polymers that act as signals for the development of nodules on legumes for fixing atmospheric nitrogen (Fisher and Long, 1992; Denarie and Cullimore, 1993); though the ability of *Rhizobiales* to degrade chitin is not well defined. The most abundant *Rhizobiales* genus in MSC-1 was *Ensifer*, which also participates in production of chitin precursor molecules, but is not known to degrade chitin based on a literature survey. Since MSC-1 was developed under chitin enrichment conditions and known chitin degraders comprise a large proportion of MSC-1, it is possible that populations that produce and degrade chitin metabolites (chitin dimers or trimers) would also emerge. This possibility is supported by observations in Figure 7 that show increased biomass production when *Streptomyces* and *Ensifer* were cultured together. Now that we have individual strains of each of these genera the collection of genomes combined with metabolic modeling will further aid in the revelation of positive interactions and specific metabolic dependencies between strains (Henry et al., 2016).

Previous studies have also shown that several species of *Streptomyces* (included in our network but with centrality values lower than others) can degrade chitin to simpler molecules (Schrempf, 1999, 2001). As the chitin molecule is too large to be directly imported into the cell, extracellular enzymes are excreted by *Streptomyces* species to degrade chitin to trimers or dimers of NAG (Beier and Bertilsson, 2013). Interestingly, although *Streptomyces* was highly abundant in MSC-1 on plates and occupied an important position in an MSC-1 network derived from incubation in soil with chitin, this genus itself was not highly abundant in the native soil when it was amended with chitin. This is in line with other studies that have amended soil with chitin but found no increase in *Streptomyces* abundance or activity (De Tender et al., 2019), despite the known ability of this bacteria to degrade chitin. This may be due to other, more bioavailable, nutrients in soil or that fact that *Streptomyces* may be using resources more slowly.

## Conclusion

Here we described the development and analysis of a model soil consortium, MSC-1, that contains several species that are found in the native soil and that were enriched to grow together on a complex carbon substrate. Importantly, we demonstrated that this consortium can be stably stored and revived. Such a consortium represents a resource that can be shared with other researchers for a variety of detailed experiments and modeling of interspecies interactions and metabolic co-dependencies between soil microorganisms during carbon decomposition. Because the soil microbiome is one of the most complex ecosystems on the planet, the outcomes of this study will aid the soil microbiology research community in understanding specific interspecies interactions that drive the soil microbiome.

## Data Availability Statement

The authors acknowledge that the data presented in this study must be deposited and made publicly available in an acceptable repository, prior to publication. Frontiers cannot accept a manuscript that does not adhere to our open data policies. Amplicon sequence data has been deposited at the PNNL DataHUB repository here: https://doi.org/10.25584/WAIsoCMSC1/1635272 and is available for download under the project accession 10.25584/WAIsoCMSC1/1635272. The version described in this article is the first version. Package includes amplicon 16S rRNA fastq files, supplementary information, MIxS_MIMARKS.soil.5.0 metadata information, R markdown, and package “Read Me” file. The R markdown processing scripts used to process the data and build graphs are available at: https://github.com/dtnaylor124/Model MicrobialCommunity.

## Author Contributions

RM designed the experiments and wrote the manuscript. DN performed the experiments and analyzed the data. YF performed the experiments. MD contributed to strain isolation. SF performed sequencing analysis. KH and JJ designed the experiments and directed the overall project. All authors contributed to the article and approved the submitted version.

## Conflict of Interest

The authors declare that the research was conducted in the absence of any commercial or financial relationships that could be construed as a potential conflict of interest.

## Supplementary Material

FIGURE S1 Respiration of native soil with chitin. Respiration levels of native soil grown in dark at 20°C for several months is indicted. The orange line represents respiration levels with no chitin added. The gray line represents respiration with chitin added to 10 ppm. The yellow line represents respiration with chitin added to 50 ppm. The blue line represents respiration with chitin added to 100 ppm. Error bars indicate standard deviation.

FIGURE S2 Reconstitution of consortia. Phylum level representation of each consortium is shown for the original parent consortia after 22 weeks on plates (Original), and in each of 4–5 glycerol or lyophilized stocks.

FIGURE S3 Reconstitution of consortia. Class level representation of each consortium is shown for the original parent consortia after 22 weeks on plates (Original), and in each of 4–5 glycerol or lyophilized stocks.

FIGURE S4 Reconstitution of consortia. Order level representation of each consortium is shown for the original parent consortia after 22 weeks on plates (Original), and in each of 4–5 glycerol or lyophilized stocks.

FIGURE S5 Parent/stock comparisons of consortia for glycerol storage. Bray-Curtis distance is on the y-axis for the reconstituted stock, the parent community and the distance from the other six parent communities. A star indicates NOT significantly different from the reconstituted stocks (*p*-value > 0.05).

FIGURE S6 Parent/stock comparisons of consortia for lyophilization storage. Bray-Curtis distance is on the y-axis for the reconstituted stock, the parent community and the distance from the other six parent communities. A star indicates NOT significantly different from the reconstituted stocks (*p*-value > 0.05).

FIGURE S7 Reconstitution of Generation 2 of consortia. Consortia are shown on the x-axis with type shown along the top. Bray-Curtis distances are shown on the y-axis.
