# Supplementary figures and images for: Development and Analysis of a Stable, Reduced Complexity Model Soil Microbiome
Source: Front Microbiol. 2020 Aug 26;11:1987. doi: 10.3389/fmicb.2020.01987 (PMC7479069; doi:10.3389/fmicb.2020.01987)

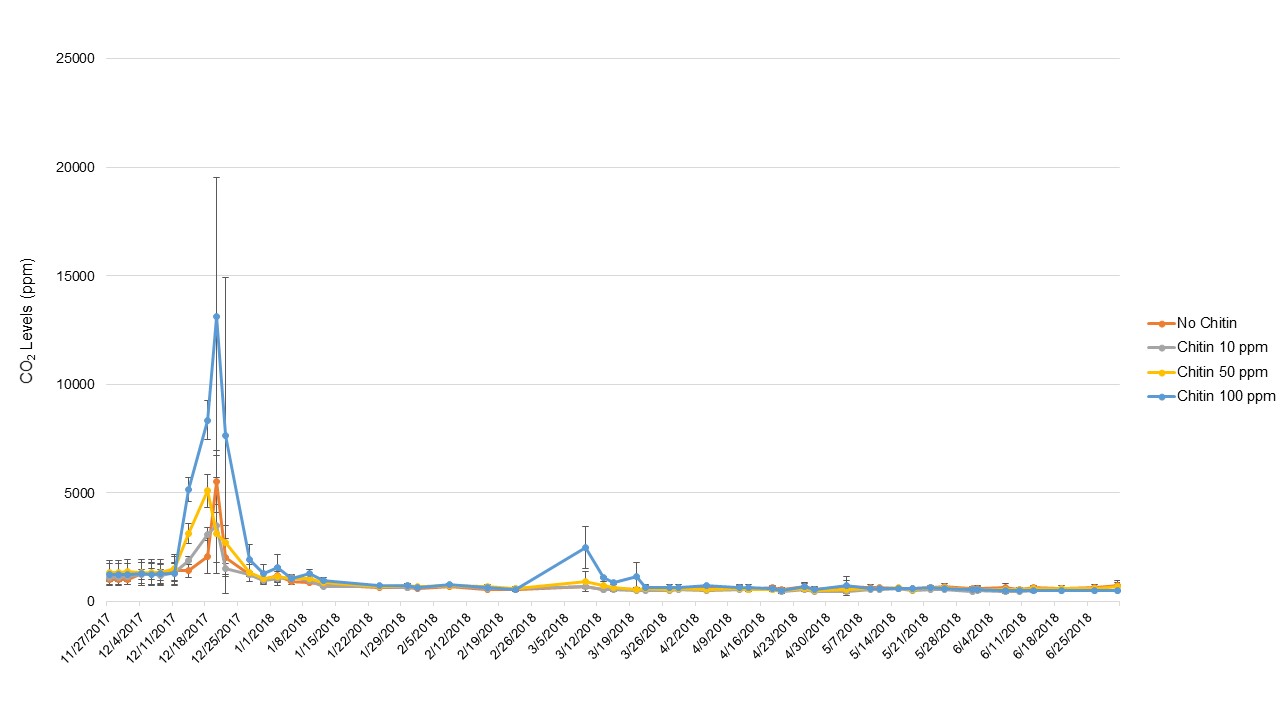

Supplement: FIGURE S1 — Respiration of native soil with chitin. Respiration levels of native soil grown in dark at 20°C for several months is indicted. The orange line represents respiration levels with no chitin added. The gray line represents respiration with chitin added to 10 ppm. The yellow line represents respiration with chitin added to 50 ppm. The blue line represents respiration with chitin added to 100 ppm. Error bars indicate standard deviation. [file Image_1.JPEG]

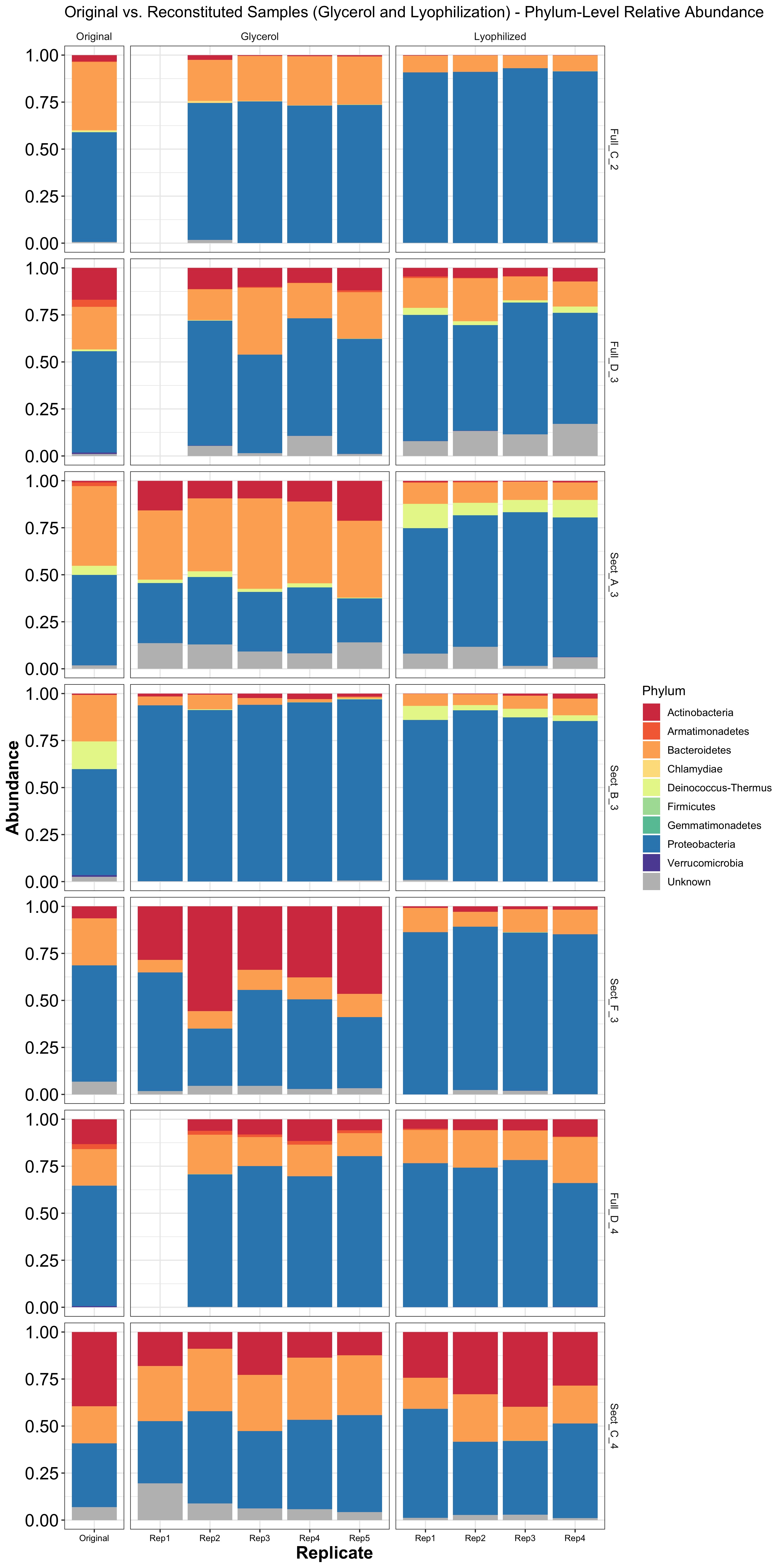

Supplement: FIGURE S2 — Reconstitution of consortia. Phylum level representation of each consortium is shown for the original parent consortia after 22 weeks on plates (Original), and in each of 4–5 glycerol or lyophilized stocks. [file Image_2.JPEG]

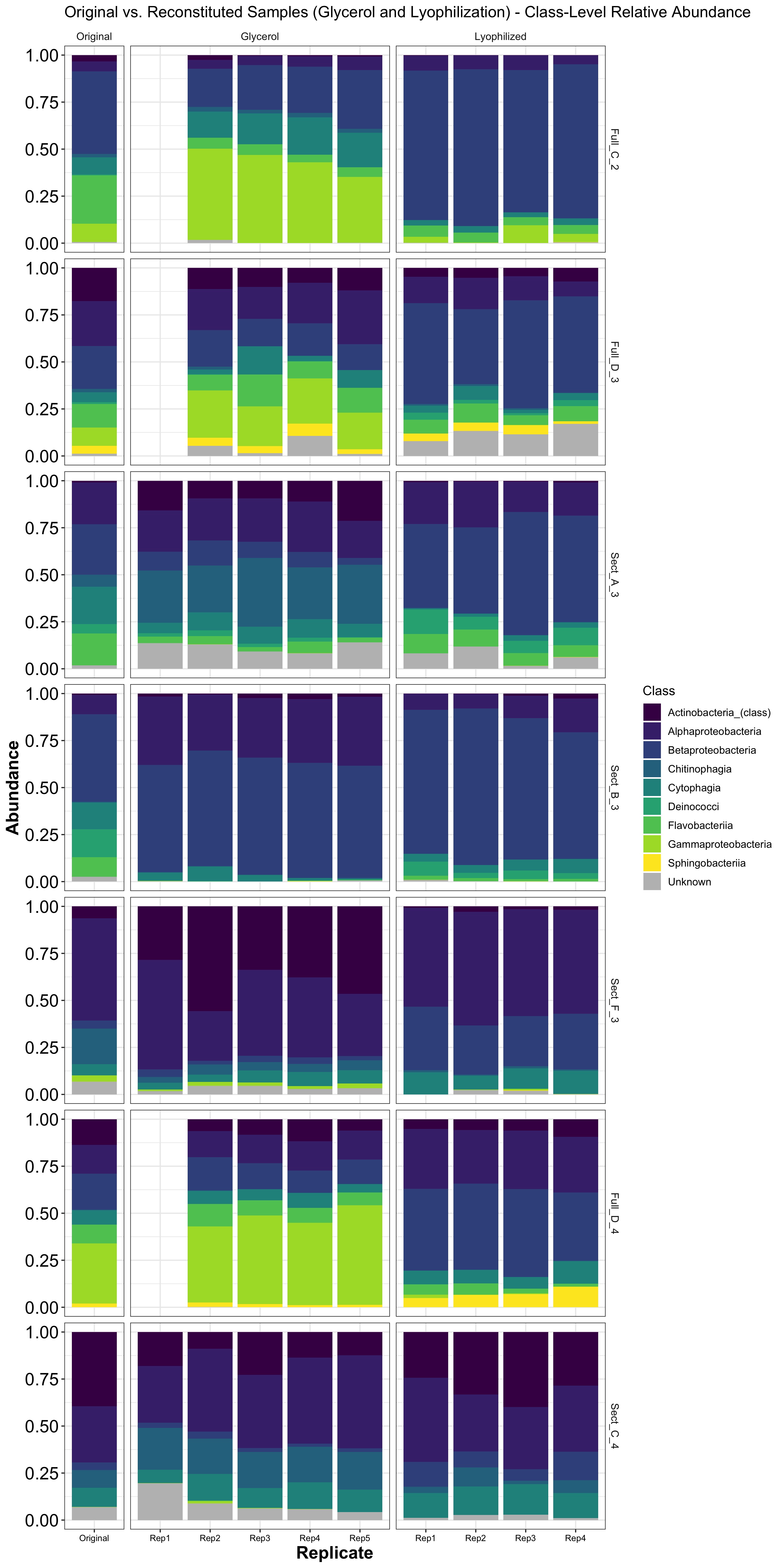

Supplement: FIGURE S3 — Reconstitution of consortia. Class level representation of each consortium is shown for the original parent consortia after 22 weeks on plates (Original), and in each of 4–5 glycerol or lyophilized stocks. [file Image_3.JPEG]

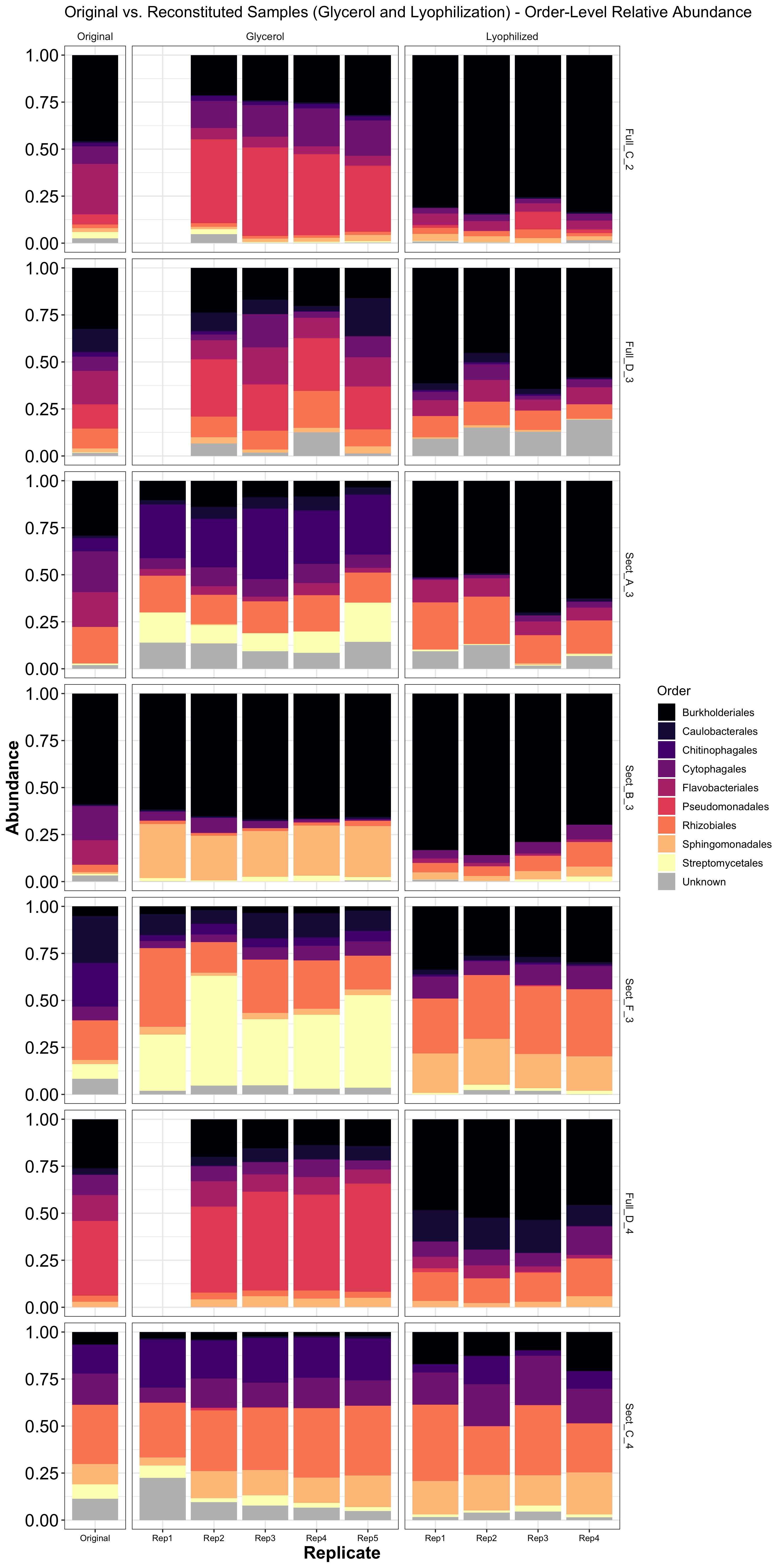

Supplement: FIGURE S4 — Reconstitution of consortia. Order level representation of each consortium is shown for the original parent consortia after 22 weeks on plates (Original), and in each of 4–5 glycerol or lyophilized stocks. [file Image_4.JPEG]

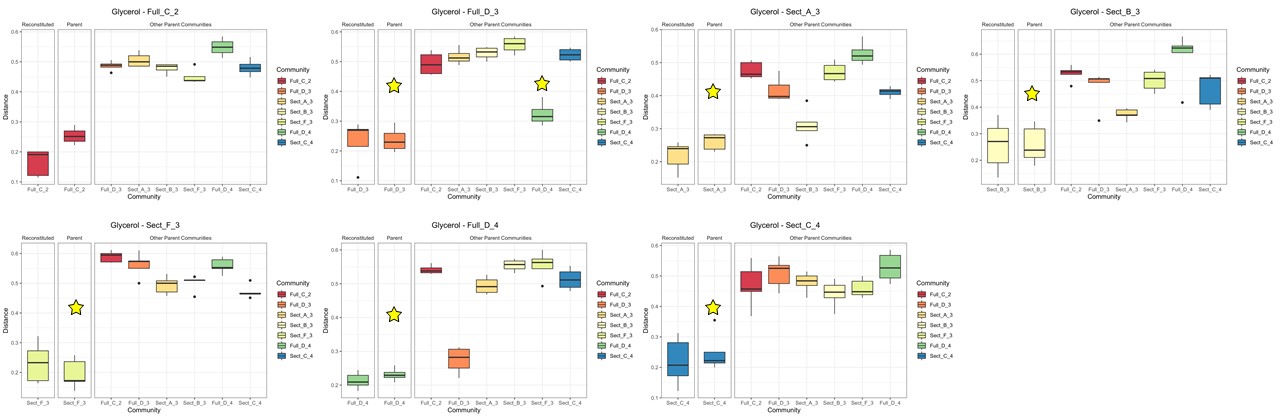

Supplement: FIGURE S5 — Parent/stock comparisons of consortia for glycerol storage. Bray-Curtis distance is on the y-axis for the reconstituted stock, the parent community and the distance from the other six parent communities. A star indicates NOT significantly different from the reconstituted stocks (p-value > 0.05). [file Image_5.jpg]

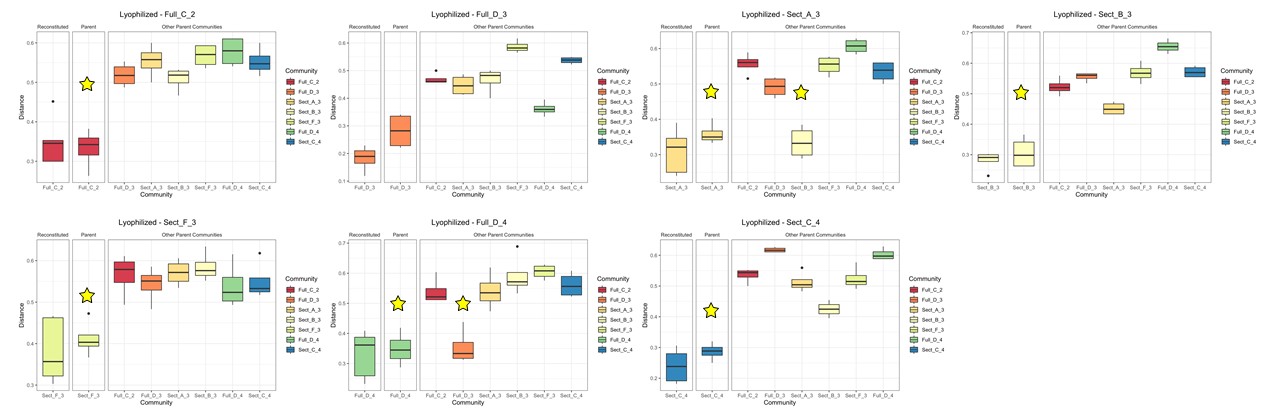

Supplement: FIGURE S6 — Parent/stock comparisons of consortia for lyophilization storage. Bray-Curtis distance is on the y-axis for the reconstituted stock, the parent community and the distance from the other six parent communities. A star indicates NOT significantly different from the reconstituted stocks (p-value > 0.05). [file Image_6.jpg]

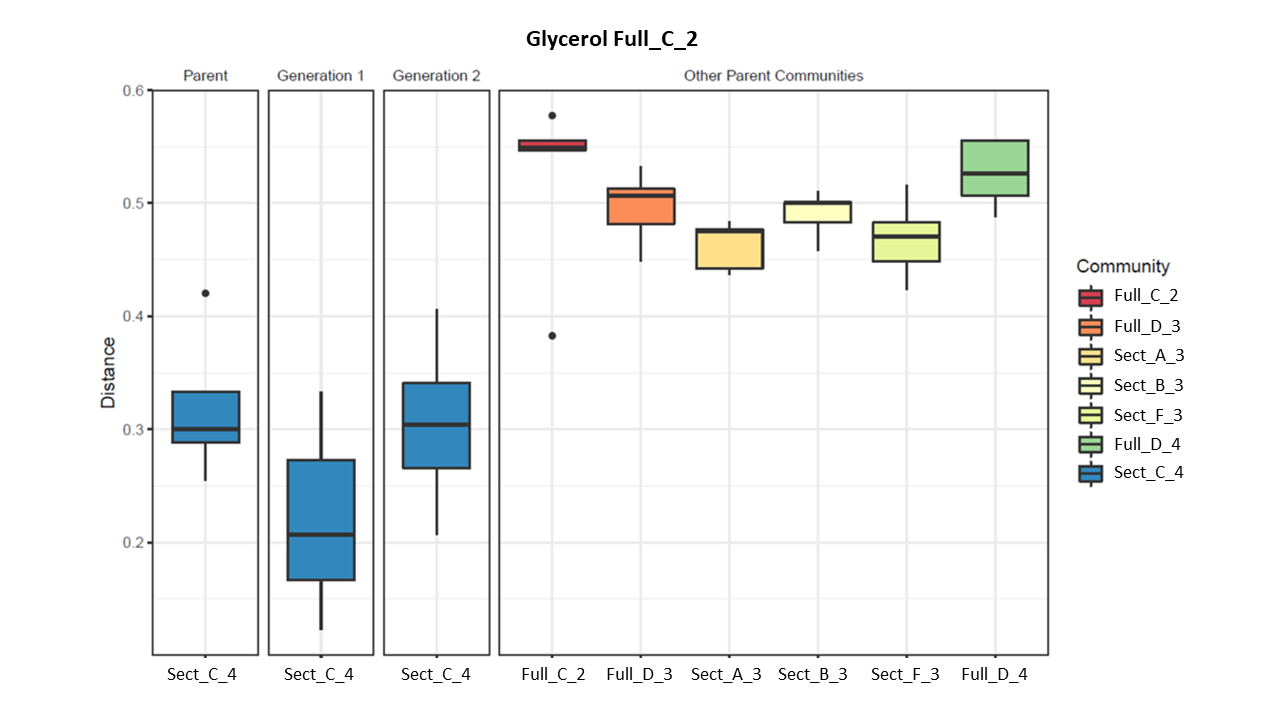

Supplement: FIGURE S7 — Reconstitution of Generation 2 of consortia. Consortia are shown on the x-axis with type shown along the top. Bray-Curtis distances are shown on the y-axis. [file Image_7.tif]
